# Effects of Hydroxytyrosol against Lipopolysaccharide-Induced Inflammation and Oxidative Stress in Bovine Mammary Epithelial Cells: A Natural Therapeutic Tool for Bovine Mastitis

**DOI:** 10.3390/antiox9080693

**Published:** 2020-08-03

**Authors:** Roberta Fusco, Marika Cordaro, Rosalba Siracusa, Alessio Filippo Peritore, Ramona D’Amico, Patrizia Licata, Rosalia Crupi, Enrico Gugliandolo

**Affiliations:** 1 Department of Chemical, Biological, Pharmaceutical and Environmental Science, University of Messina, 98166 Messina, Italy; rfusco@unime.it (R.F.); cordarom@unime.it (M.C.); rsiracusa@unime.it (R.S.); aperitore@unime.it (A.F.P.); rdamico@unime.it (R.D.); egugliandolo@unime.it (E.G.); 2 Department of Veterinary Science, University of Messina, 98168 Messina, Italy

**Keywords:** hydroxytyrosol, oxidative stress, inflammation, bovine mastitis

## Abstract

Background: Bovine mastitis is a growing health problem, affecting both welfare of dairy cattle and milk production. It often leads to chronic infections, disturbing the quality of milk and resulting in cow death. Thus, it has a great economic impact for breeders. Methods: In this study, we evaluated the protective effect of hydroxytyrosol—a natural molecule which is the major constituent of many phyto-complexes—in an in vitro model of mastitis induced by LPS (1μg/mL). Results: Our results showed that hydroxytyrosol (10 and 25 μM) was able to prevent the oxidative stress induced by LPS (intracellular ROS, GSH and NOX-1) and the consequently inflammatory response (TNF-α, IL-1β and IL-6). The protective effect of hydroxytyrosol is also related to the enhancement of endogenous antioxidant systems (Nrf2, HO-1, NQO-1 and Txnrd1). Moreover, hydroxytyrosol showed an important protective effect on cell functionality (α-casein S1, α-casein S2 and β-casein). Conclusions: Taken together, our results showed a significant protective effect of hydroxytyrosol on oxidative stress and inflammatory response in MAC-T cells. Thus, we indicated a possible important therapeutic role for hydroxytyrosol in the prevention or management of bovine mastitis.

## 1. Introduction

Bovine Mastitis is characterized by the inflammation of the mammary gland that occurs in response to several different stimuli, such as microorganism infection [1]. The udders of infected cows are one of the major reservoir of contagious pathogens. During milking they spread from cow to cow, inducing chronic subclinical infections. Contagious pathogens include: Streptococcus agalactiae, Staphylococcus aureus, *Corynebacterium bovis* and *Mycoplasma* spp. [2]. Differently, environmental mastitis is defined as intramammary infections caused by pathogens of the environment in which the cow lives [3]. Environmental pathogens include *Klebsiella* spp., *Escherichia coli*, *Streptococcus uberis* and *Streptococcus dysgalactiae*. Most those infections are characterized by a short duration [4].

Moreover, mastitis influences yield, composition and quality of the milk produced by the affected cow and represents a serious economic issue for the farmer [5]. In particular, it often leads to culling of chronically infected cows and occasional deaths [6]. The use of antibiotics is a consolidate pharmacological approach for mastitis treatment that results in several side effects [7]. New studies into pharmacological approaches for the treatment of this pathology is required for reducing the use of antibiotics that are dangerous for health of consumers and antibiotic resistance spread [7,8]. Nowadays, several natural molecules or derivates has been tested for the treatment of bovine mastitis [9]. Natural molecules are preferred thanks to their safer use for both the animal and its derivatives [10,11,12]. Interesting results have been obtained from antioxidant natural compounds such as baicalein [13], moringa extract [14] and curcumin [15].

There is a well consolidated crosstalk between inflammation and oxidative stress. Recent evidences have shown the beneficial effects of the antioxidant therapy in several pathologies [8,16,17,18,19,20,21,22]. A key role for oxidative stress in bovine mastitis is widely recognized: dietary supplementation with antioxidant nutrients has shown protective action and improvement of immune resistance against infections [23,24]. Oxidative stress in early lactation cows plays a central role in dysfunctional inflammatory response [25]. Additionally, during the periparturient period there is an increase in the production of the reactive oxygen species. The free radicals produced induce a positive feedback which can further aggravate the pathologic condition [26]. Hydroxytyrosol (3,4-dihydroxyphenylethanol, HT) is a polyphenol mainly present in the fruit and in the leaf of *Olea europaea* L., a plant belonging to the Oleaceae family. This plant family comprises numerous different species distributed throughout the Mediterranean basin. HT is also the main component of extra virgin olive oil: it is present in esterified or free form and it represents the 70–80% of the total phenolic fractions [27]. High HT contents have been found in the olive leaf extract and in olive mill waste water, making it a potentially useful waste product [28]. Several works describe the antioxidant properties of HT [29]: its antioxidant power is two times higher than coenzyme Q10 and ten times higher than green tea [30]. Additionally, numerous biologic activities have been ascribed to HT, although in some cases its mechanism of action has not been clarified. Of particular interest are the anti-inflammatory and antimicrobial properties of HT, which have been described in many diseases [28,31,32,33,34,35,36]. In particular, it has been reported that HT was able to inactivate the staphylococcal enterotoxin A, a key factor in the patho-physiology of animal mastitis [37]. Therefore, the application of HT in the treatment or prevention of bovine mastitis would be an interesting therapeutic strategy.

Bovine mammary epithelial cell lines, like mammary alveolar (MAC-T) cells, are one of the most used cell lines to study inflammation, lipid metabolism and apoptosis [38,39,40,41]. In particular, MAC-T cells represent a convenient validation system for investigating lactation and bioactive substances with the mammary tissue-specific casein promoter [42]. For this process to happen, the mammary gland grows and differentiates through extensive tissue remodeling. When treated with prolactin, insulin and retinoic acid this cell line differentiates into β-casein–secreting cells [43]. In this study, we investigated the effect of HT on lipopolysaccharide-induced inflammation and oxidative stress in bovine mammary epithelial (MAC-T) cells.

## 2. Materials and Methods

### 2.1. Cell Culture

Bovine mammary epithelial cell line (MAC-T cells) [44], were cultured in DMEM medium containing 10% fetal bovine serum (FBS) and 200 U/mL streptomycin/penicillin (Sigma-Aldrich, Milano, Italy) and incubated at 37 °C in a humidified atmosphere containing 5% CO_2_. The medium was changed every 48 h. The cells were split at 80–90% confluency using 0.25% trypsin solution.

### 2.2. Cell Differentiation

MAC-T cell were detached with 0.25% of trypsin-ethyl-enediaminetetraacetic acid (Sigma-Aldrich, Milano, Italy). The initial number of cells in each group was 5 × 10^4^ cells/well in a 6-well plate and cultured for 4 days, and then the cells in each group were split on additional 6-well plates on 4 and 5 days. MAC-T cells were differentiated as already described [45,46]. Briefly, cells were cultured (5 × 104 cells/well in 6-well plate) in serum-free DMEM for 16 h and then cultured in high-glucose DMEM addition of 5% FBS, 5-μg/mL insulin (Sigma, USA), 1-μg/mL hydrocortisone (Sigma, USA), 5-μg/mL prolactin (PRL) (Sigma, USA) and 1-μM retinoic acid (RA) (Sigma, USA). During this period, the medium was changed every 24 h. To determine casein mRNA expression, 10-μM and 25-μM HT was added to cells 1 h before LPS-stimulation.

### 2.3. Cell Treatment

Cell were pretreated with HT (10 μM and 25 μM) (Sigma-Aldrich, Milano, Italy). One hour after HT pretreatment, cells were stimulated with LPS 1 μg/mL (*Escherichia coli* O111:B4, Sigma-Aldrich, Milano, Italy) for 1 or 6 h as previously described [47]. LPS concentrations were chosen based on previous studies by others using mammary epithelial cells [48,49].

### 2.4. Cell Viability Assay

The possible toxic effect of HT on MAC-T cells was determined by methyl thiazolyl tetrazolium (MTT) assay as previously described [50]. Briefly, cells seeded in a 96-well plate and incubated with HT at 10, 25, 50, 100, 250 μM, for 24 h in a 96-well plate, followed by the MTT treatment (10 μL of 0.5 mg/mL) for 4 h. Acidic isopropanol was added to dissolve any deposited formazan. The optical density at 550 nm was measured using a microplate reader and used to calculate the cell viability.

### 2.5. Western Blot Analysis

Cells were lysed with a hypotonic buffer solution containing 20 mM Tris (pH 7.4), 10 mM NaCl, 3 mM MgCl2 and a protease inhibitor mixture. After addition of 10% Triton-X 100, cell lysates were centrifuged at 650× *g* for 10 min at 4 °C and supernatants were collected as cytosolic fractions. Remaining pellets were resuspended in cell extraction buffer [100 mM Tris (pH 7.4), 1% Triton X-100, 10% glycerol and 0.1% SDS] containing protease inhibitor mixture. Homogenates were then centrifuged at 14,000× *g* for 20 min at 4 °C and supernatants were collected as nuclear fractions. The levels of nuclear factor erythroid 2-related factor 2 (Nrf2) quantified in nuclear fraction. The filters were blocked with 1 × PBS, 5% (*w*/*v*) nonfat dried milk (PM) for 40 min at room temperature and subsequently probed with specific Nrf2 antibody (Novus biologicals, Centennial, CO, USA) in 1 × PBS, 5% *w/v* nonfat dried milk, 0.1% Tween-20 (PMT) at 4 °C, overnight. Membranes were incubated with peroxidase-conjugated goat anti-rabbit IgG (1:2000, Jackson Immuno Research, West Grove, PA, USA) at room temperature for 1 h. Laminin protein were used as internal standard for nuclear extracts. Signals were detected with enhanced chemiluminescence detection system reagent according to manufacturer’s instructions (Super-Signal West Pico Chemiluminescent Substrate, Pierce). The relative expression of the protein bands was quantified by densitometry with Bio-Rad (Bio-Rad, Milan, Italy) ChemiDoc XRS software and standardized to lamin levels. A preparation of commercially, molecular weight markers made of proteins of molecular weight 10–250 kDa was used to define molecular weight positions and as reference concentrations for each molecular weight.

### 2.6. Oxidative Stress Markers

In order to evaluate total cellular reactive oxygen species (ROS) we employed the 2′,7′-dichlorodihydrofluorescein diacetate (H2DCFDA) dye. MAC-T cells were grown to confluence in 6-well plates, trypsinized and then washed twice with 1x-washing buffer. Subsequently, cells were incubated with 1-μM H2DCFDA dye at 37 °C. The fluorescence microplate reader detected the light emission. The levels of increased ROS production were expressed as percentage of the control (nmol/mL). Reduced GSH was measured by a commercially available kit (EIAGSHC, Thermo Fisher Scientific, Milan, Italy).

### 2.7. ELISA

Secretion of TNF-α, IL-6 and IL-1β were measured using commercial ELISA kits from R&D Systems, Minneapolis, MN, USA. Briefly, after the already described treatment, 100 μL of samples or standards were added in each well and incubated for 2 h at room temperature. After 2 washes, 100 μL of the diluted detection antibody was added to each well and incubated for 2 h at room temperature. After 2 washes, 100 μL of the working dilution of streptavidin–HRP A was added to each well and incubated for 2 h at room temperature. After washes, 100 μL of substrate solution was added to each well and incubated for 20 min at room temperature in the dark. The reaction was then stopped by adding 50 μL of stop solution. Absorbance at 450 nm was recorded to calculate protein concentration.

### 2.8. Real-Time PCR

To evaluate the mRNA expression of target genes, RNA was extracted from MAC-T cells using RNeasy kit (Qiagen, Milan, Italy), for real-time polymerase chain reaction (PCR) analysis. Briefly, samples were first lysed and then ethanol was added to provide ideal binding conditions. The lysates were then loaded into the RNeasy silica membrane. RNA binds and all contaminants were efficiently washed away. The residual amounts of DNA remaining were removed using a convenient on-column DNase treatment. Pure, concentrated RNA was eluted in 50 µL water. iScript RT-PCR kit (Bio-Rad) was used to synthesize first-strand cDNA. Briefly, the reverse transcription master mix was prepared adding to 1 µg of RNA template the iScript RT Supermix (5x RT supermix with RNase H+ Moloney (gray cap, 25 or 100 reactions) murine leukemia virus (MMLV) reverse transcriptase, RNase inhibitor, dNTPs, oligo(dT), random primers, buffer, MgCl2 and stabilizers) and the nuclease-free water. The complete reaction mix was incubated in a thermal cycler (Priming 5 min at 25 °C, Reverse transcription 20 min at 46 °C, RT inactivation for one minute at 95 °C). Real-time PCR analysis was performed by SYBR Green method on a StepOnePlus real-time PCR system (Applied Biosystems, Waltham, MA, USA). PCR conditions were as follows: initial denaturation at 95 °C for 15 min, followed by 45 cycles of amplification at 95 °C for 20 s and 60 °C for 40 s. Final extension at 60 °C for 60 s and a hold at 4 °C were then performed. Data analysis was performed using the 2−ΔΔCt method, in which relative mRNA expression of target mRNAs [NADPH oxidase-1 (NOX-1), heme oxygenase-1 (HO-1), NAD(P)H: quinone oxidoreductase-1 (NQO-1) and thioredoxin reductase 1 (Txnrd1) and casein isoforms] was compared to that of a constitutively expressed gene (i.e., GAPDH). Primer sequences used in this study were: NADPH oxidase-1 (NOX-1): (Bio-Rad, Italy), HO-1: (forward) AGG ATT TGT CAG AGG CCC TGA A (reverse) CAA AGA CGC CAT CAC CAG CTT A, NQO-1: (forward) GGT GCT CAT AGG GGA GTT CG (reverse) GGG AGT GTG CCC AAT GCT AT, Txnrd1: (forward) CGG TAT TGC TGG CAA TAG GAA GAG (reverse) GGC ATA GAT GTA AGG CAC GTT GGT, α-casein S1: (forward) GGG AAT CCAT GCC CAA CAG AAA GA (reverse) GGA ACG TAA TAC CAG GCA CCA GAT, α-casein S2: (forward) GGA CGA TAA GCA CTA CCA GAA AGC (reverse) AGA GTG GGA GTA ATG GGA ACA GCA, β casein: (forward) CCT AAC AGC CTC CCA CAA AA (reverse) AGA CTG GAG CAG AGG CAG AG, GAPDH (forward) ATG ATT CCA CCC ACG GCA AGT T (reverse) ACC ACA TAC TCA GCA CCA GCA T. The results are expressed as fold-changes [51].

### 2.9. Statistical Analysis

For each experiment, three or more independent experiments were performed and for each experiment five repeat samples were used. The data resulting from all experiments are expressed as means ± SEM. Statistical differences between groups were compared using ANOVA, followed by Tukey’s test. A *p*-value of less than 0.05 was considered statistically significant.

## 3. Results

### 3.1. HT Effect on MAC-T Cell Viability

**Figure 1:**
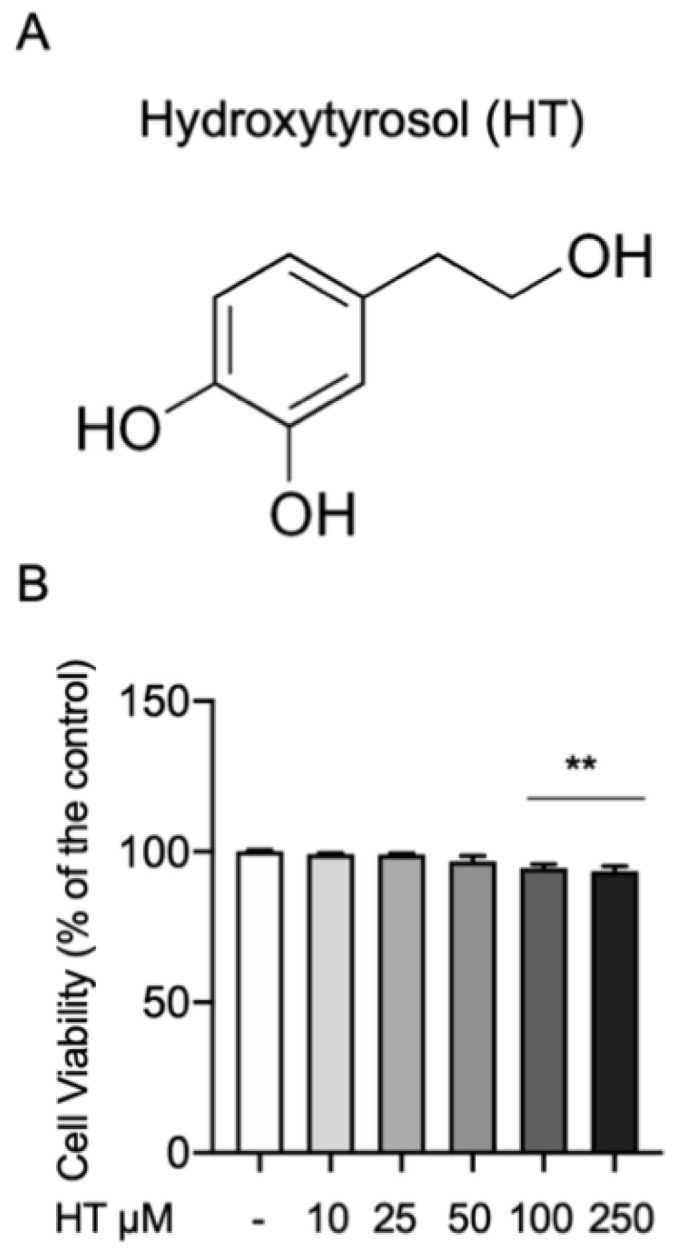
(**A**) Chemical structure of hydroxytyrosol and (**B**) effect on cell viability MTT assay. Data representative of at least three experiments, means ± SEM; ** *p* < 0.01 vs. control.

First, we investigated any toxic effect of HT on the MAC-T cells by the MTT cell viability assay. The concentrations of HT evaluated were 10, 25, 50, 100 and 250 μM. Cells were incubated with HT for 24 h and then treated with MTT (10 μL of 0.5 mg/mL) for 4 h. As show in Figure 1, no statistical differences were detected at 10-, 25- and 50-μM HT (respectively 99.2% ± 0.37; 99% ± 0.44; 96.8% ± 1.77) than the control. We found a significant reduction in cell viability at the concentration of 100 (94.6% ± 1.2) and 250 μM (93.3% ± 1.56) (Figure 1B and Table S1 in Supplemental Material).

### 3.2. Protective Effect of HT in LPS Induced Oxidative Stress in MAC-T Cell

**Figure 2:**
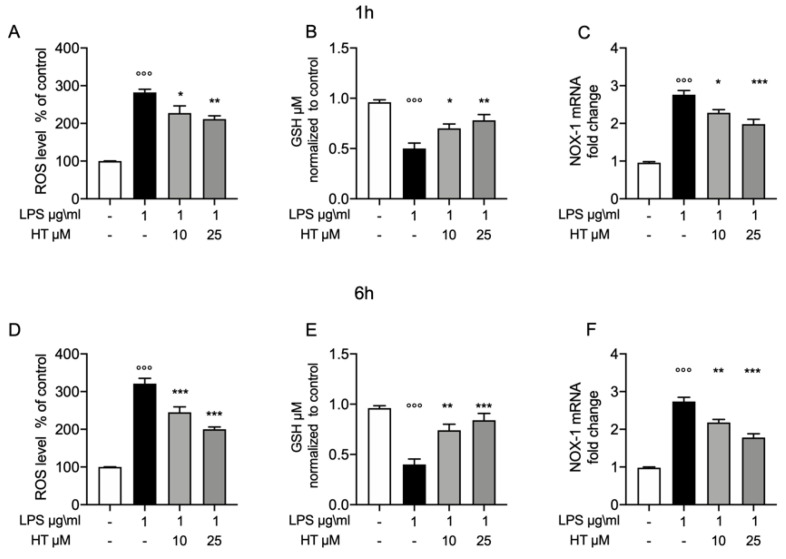
Protective effect of hydroxytyrosol (HT) in LPS (1 μg/mL) induced oxidative stress in MAC-T cell. (**A**) Intracellular ROS, (**B**) GSH levels and (**C**) NOX-1 mRNA levels, one hour post stimulation LPS; (**D**) intracellular ROS, (**E**) GSH levels and (**F**) NOX-1 mRNA levels, six hours post LPS. Data representative of at least three experiments, means ± SEM; °°° *p* < 0.001 vs. control; * *p* < 0.05 vs. LPS; ** *p* < 0.01 vs. LPS; *** *p* < 0.001 vs. LPS.

To test whether the protective effect of HT in LPS induced oxidative stress, we pretreated the MAC-T cell with HT (10-μM and 25 μM) for 1 h and then stimulated them with LPS 1-μg/mL for 1 h and 6 h. One hours after LPS-stimulation increased oxidative stress was detected, as shown by the increased levels of intracellular ROS (Figure 2A), reduction in GSH levels (Figure 2B), than control (282 ± 8.6 vs. 100 ± 0.37 for ROS and 0.5 ± 0.05 vs. 0.95 ± 0.02 for GSH, respectively). This trend was confirmed by the upregulation of the NADPH oxidase-1 (NOX-1) mRNA level (Figure 2C), than control (2.76 ± 0.11). HT treatment (10 μM and 25 μM) reduced in a dose dependent manner the cellular oxidative stress by reducing NOX-1 mRNA expression (HT 10 μM: 2.28 ± 0.08) (HT 25 μM: 1.98 ± 0.12) (Figure 2C) and ROS intracellular levels (HT 10 μM: 227.4 ± 19.02 and HT 25 μM: 211. ± 8.87) (Figure 2A) and increasing GSH levels (HT 10 μM: 0.7 ± 0.04 and HT 25 μM: 0.78 ± 0.05) (Figure 2B) (Table S2 in Supplemental Material). Additionally, we evaluated the same parameters six hours after LPS-stimulation. LPS increased ROS and GSH levels than control (321 ± 14.18 vs. 100 ± 0 for ROS and 0.4 ± 0.05 vs. 0.96 ± 0.02 for GDH, respectively) (Figure 2D,E, respectively). HT treatment was able to reduce intracellular ROS levels (HT 10 μM: 245 ± 14.49 and HT 25 μM: 200.2 ± 6) and increase GSH levels (HT 10 μM: 0.74 ± 0.06 and HT 25 μM: 0.84 ± 0.06). RT-PCR analysis for NOX-1 mRNA expression showed increased levels in LPS treated cells than control (2.74 ± 0.10) (Figure 2F). HT treatment at 10 μM and 25 μM reduced NOX-1 mRNA expression (HT 10 μM: 2.18 ± 0.08 and HT 25 μM: 1.78 ± 0.10) (Figure 2F and Table S3 in Supplemental Material).

### 3.3. Protective Effect of HT in LPS Induced Inflammatory Response in MAC-T Cell

**Figure 3:**
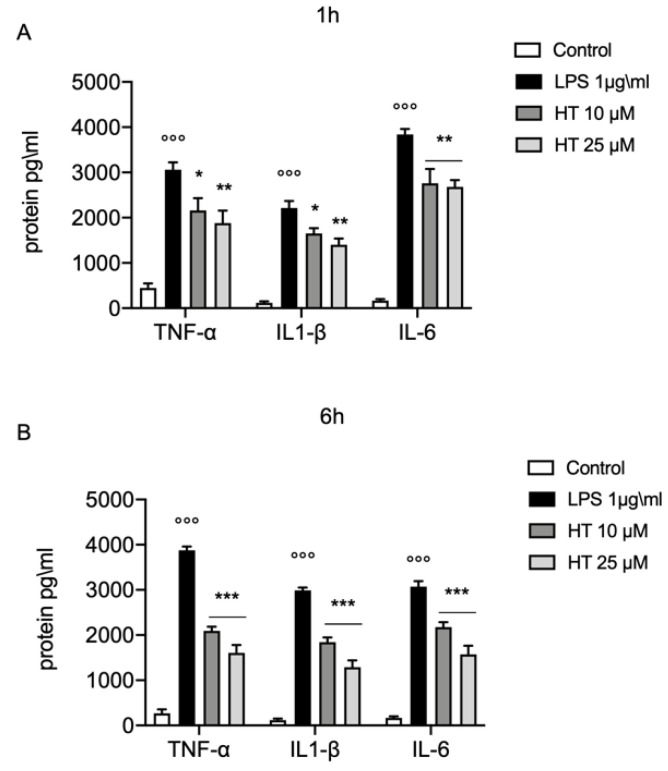
Protective effect of HT in LPS induced inflammatory response in MAC-T cell. (**A**) TNF-α, IL-1β and IL-6 levels one hour after LPS-stimulation 1 μg/mL; (**B**) TNF-α, IL-1β and IL-6 levels six hours after LPS-stimulation 1 μg/mL. Data representative of at least three experiments, means ± SEM; °°° *p* < 0.001 vs. control; * *p* < 0.05 vs. LPS; ** *p* < 0.01 vs. LPS; *** *p* < 0.001 vs. LPS.

Increased in oxidative stress is closely related with the inflammatory response. One hour after LPS-stimulation (1 μg/mL) we found a significant increased levels of the main inflammatory cytokines TNF-α (3060 ± 163.1 vs. 450 ± 97.47), IL-1β (2214 ± 154.9 vs. 118 ± 34.26) and IL-6 (3840 ± 120.8 vs. 170 ± 30) (Figure 3A). The treatment with HT at the concentration of 10 and 25 μM significant prevent the increase of TNF-α (HT 10 μM: 2160 ± 271.3 and HT 25 μM: 1880 ± 276.4), IL-1β (HT 10 μM: 1650 ± 120.4 and HT 25 μM: 1400 ± 138.7) and IL-6 levels (HT 10 μM: 2760 ± 317.2 and HT 25 μM: 2720 ± 171.5) (Figure S3A and Table S2 in Supplemental Material). The levels of TNF-α, IL-1β and IL-6 were still significantly higher than the control group, six hours after LPS-stimulation (3876 ± 85.88 vs. 270 ± 86.02 for TNF-α, 2988 ± 63.98 vs. 118 ± 34.26 for IL-1β, 3074 ± 119.6 vs. 170 ± 30 for IL-6) (Figure 3B). Where compared to LPS (1 μg/mL) the treatment with HT at 10 μM and 25 μM significant inhibited the increase in TNF-α (HT 10 μM: 2092 ± 92.27 and HT 25 μM: 1608 ± 174.3), IL-1β (HT 10 μM: 1842 ± 105.4 and HT 25 μM: 1288 ± 151) and IL-6 expression (HT 10 μM: 2179 ± 104.9 and HT 25 μM: 1572 ± 192.8) (Figure 3B and Table S3 in Supplemental Material).

### 3.4. Protective Effect of HT in LPS Induced Oxidative Stress in MAC-T Cell

**Figure 4:**
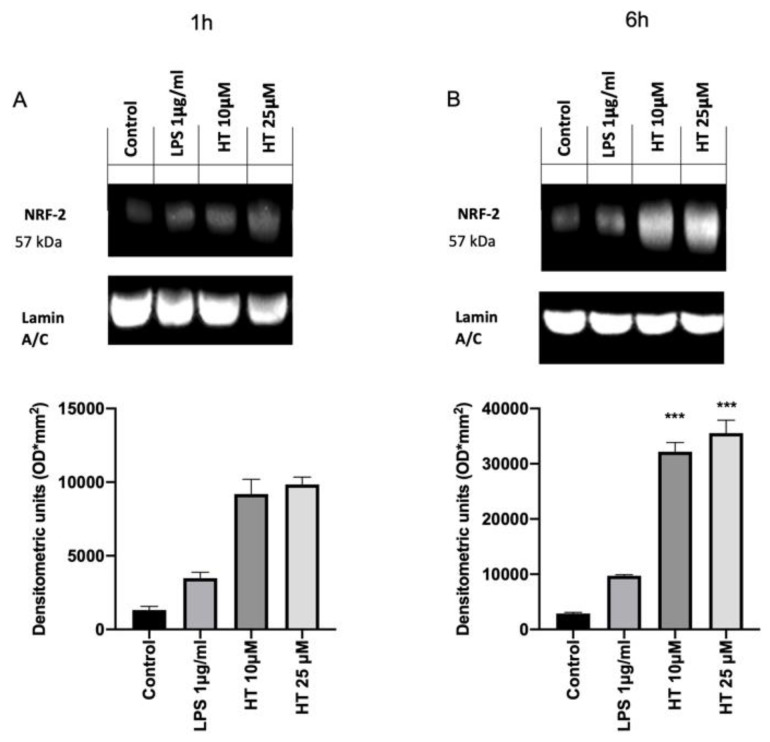
(**A**) Effect of HT on NRF2 expression: western blot analysis of NRF2 1 h after LPS 1 μg/mL stimulation and 10-μM and 25-μM HT treatment; (**B**) western blot analysis of NRF2 6 h after LPS 1 μg/mL stimulation and 10-μM and 25-μM HT treatment. Data representative of at least three experiments, means ± SEM; *** *p* < 0.001 vs. LPS.

The inflammation and oxidative stress induced by LPS-stimulation is closely associated with the activation of the Nrf2/Keap1 system and the antioxidant genes that it regulates. In order to evaluate the effect of the HT treatment on the activation of the Nrf2 pathway western blot analysis were conducted. Basal Nrf2 expression was detected in the control group (1,322,619 ± 250) (Figure 4A). Already after 1 h from LPS-stimulation Nrf2 expression were increased (3,375,711 ± 500) and HT treatment at both concentrations (10 μM and 25 μM) upregulated its expression (HT 10 μM: 9,191,217 ± 1000 and HT 25 μM: 9,849,095 ± 500) (Figure 4A). After six hours from LPS induction the Nrf2 expression was found upregulated than the control (9,703,125 ± 200 vs. 2,898,569 ± 200) and HT (10 μM and 25 μM) augmented its expression (HT 10 μM: 32,167,176 ± 166,666 and HT 25 μM: 35,553,255 ± 233,333) (Figure 4B).

### 3.5. Antioxidant Effects of HT in LPS Induced Oxidative Stress in MAC-T Cell

**Figure 5:**
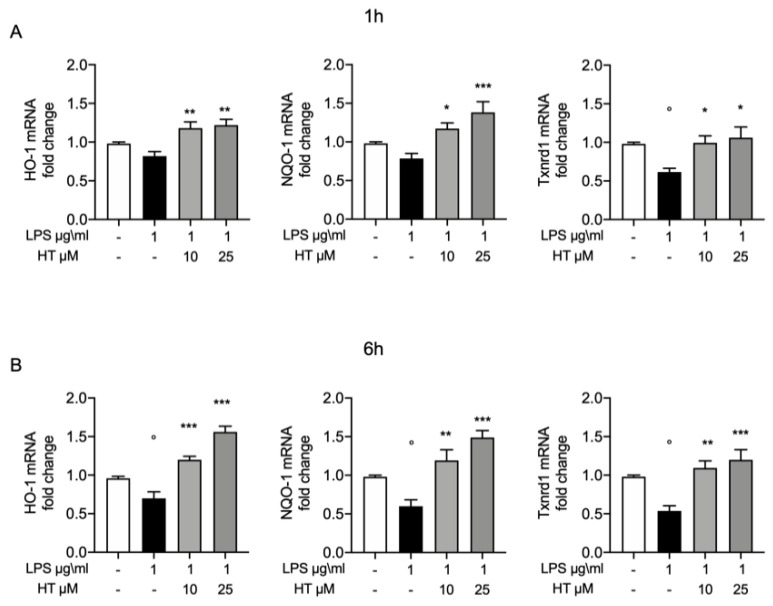
(**A**) Protective effect of HT in LPS induced oxidative stress in MAC-T cell: mRNA levels of HO-1, NQO-1 and Txnrd1 one hour post LPS 1 μg/mL stimulation and 10-μM and 25-μM HT treatment; (**B**) mRNA levels of HO-1, NQO-1 and Txnrd1 six hours post LPS 1-μg/mL stimulation and 10-μM and 25-μM HT treatment. Data representative of at least three experiments, means ± SEM; ° *p* < 0.05 vs. control; * *p* < 0.05 vs. LPS; ** *p* < 0.01 vs. LPS; *** *p* < 0.001 vs. LPS.

To further confirm the protective effects of HT on oxidative stress, we evaluated the expression of some of the endogenous antioxidant systems. In particular, we investigated by RT-PCR the mRNA expression of heme oxygenase-1 (HO-1), NAD(P)H quinone oxidoreductase-1 (NQO-1) and thioredoxin reductase 1 (Txnrd1). Already after 1 h from LPS-stimulation HO-1, NQO-1 and Txnrd1 mRNA expression were reduced (0.82 ± 0.05, 0.78 ± 0.06, 0.61 ± 0.04, respectively) and HT treatment at both concentrations was able to increase them expressions (HT 10 μM: 1.18 ± 0.08, 1.17 ± 0.07 and 0.99 ± 0.09, respectively; HT 25 μM: 1.22 ± 0.07, 1.38 ± 0.13 and 1.06 ± 0.14, respectively) (Figure 5A and Table S2 in Supplemental Material). Six hours after LPS-stimulation, the HO-1, NQO-1 and Txnrd1 mRNA levels were significantly reduced (0.7 ± 0.08, 0.59 ± 0.08 and 0.53 ± 0.06, respectively) (Figure 5B). The 10-μM and 25-μM HT treatment significantly improve the endogenous antioxidant capacity MAC-T cells by increasing in a dose dependent manner HO-1, NQO-1 and Txnrd1 mRNA expression (HT 10 μM: 1.2 ± 0.04, 1.19 ± 0.13 and 1.09 ± 0.09; HT 25 μM: 1.56 ± 0.07, 1.48 ± 0.08 and 1.2 ± 0.13, respectively) (Figure 5B and Table S3 Supplemental Material).

### 3.6. Protective Effect of HT on Casein Stimulation in LPS Stimulated MAC-T Differentiated Cell

**Figure 6:**
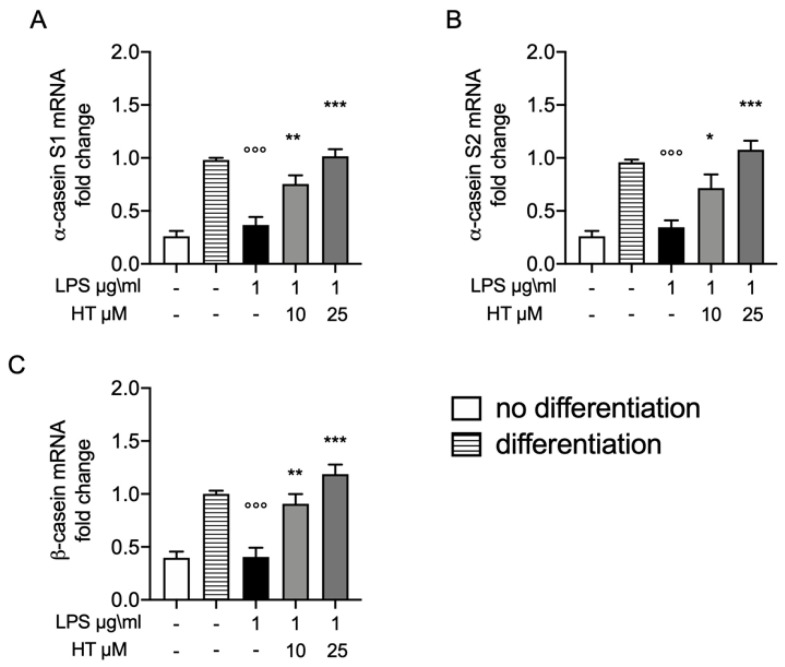
(**A**) Protective effect of HT on casein stimulation in LPS stimulated MAC-T differentiated cell: mRNA levels of α-casein S1; (**B**) α-casein S2 and (**C**) β-casein. Data representative of at least three experiments, means ± SEM; °°° *p* < 0.001 vs. control differentiated; * *p* < 0.05 vs. LPS; ** *p* < 0.01 vs. LPS; *** *p* < 0.001 vs. LPS.

Synthesis of milk is one of major complication in bovine mastitis. In order to evaluate the protective effect of HT on this parameter we evaluate the mRNA expressions of casein, including αS1, αS2 and β genes in MAC-T differentiated cell. The levels of α-casein S1, α-casein S2 and β casein 12 h after LPS-stimulation were significantly reduced (0.37 ± 0.07, 0.34 ± 0.06 and 0.40 ± 0.08, respectively) (Figure 6A–C, respectively). While HT pre-treatment 10 μM and 25 μM significantly antagonized this reduction in a dose dependent manner (HT 10 μM: 0.75 ± 0.08, 0.71 ± 0.12 and 0.90 ± 0.09; HT 25 μM: 1.01 ± 0.06, 1.07 ± 0.08 and 1.18 ± 0.09) (Figure 6A–C and Table S4 in Supplemental Material).

## 4. Discussion

Mastitis is widespread clinical disease in livestock and humans induced by microbial infection. The most used therapy to treat this pathology are the antibiotics, but they are related with significant side effects [52]. Investigating new therapeutic tools with fewer side effects is an important goal for the research. In this study, we showed for the first time the that HT, a well-known antioxidant natural compound, significantly reduced the LPS-induced inflammatory response in MAC-T cells. Additionally, we showed that HT treatment was able to increase the expression of α-casein S1, α-casein S2 and β casein in MAC-T differentiated cells after LPS-stimulation.

Recently, it has been demonstrated HT attenuate oxidative stress (glutathione (GSH/GSSH), γ-glutamylcysteine ligase activity, reactive oxygen species and malondialdehyde (MDA) production) and inflammatory response (TNF-α, IL-1β, IL-6 and IL-10) in bovine mammary epithelial cell line (BME-UV1) [53]. First of all, we tested the any toxic effect of HT on MAC-T cells by the MTT assay, based on the metabolic activity of the cells. A significant reduction in cell viability was observed at the concentration of 100 and 250 μM. Recent papers also described the HT toxicity at these concentrations and ascribed it to the HT pro-apoptotic action [29,54]. Once tested the good safety of HT, we moved to investigate its effect on the oxidative stress induced by LPS-stimulation. Previous studies shown the key role of the oxidative stress in mastitis. Overproduction of free oxygen radicals is an early event in answer to bacterial infection or LPS in MAC-T cells. Our results displayed increased oxidative stress as shown by the upregulated intracellular ROS expression and the reduced GSH level. HT pretreatment at 10 μM and 25 μM was able to prevent the increase in oxidative stress in a dose dependent manner. Additionally, we showed that HT reduced NOX-1 mRNA expression. The upregulation of this mitochondrial protein has been linked with the ROS overproduction [55]. Thus, HT pretreatment mitigated LPS-induced NOX-1 expression and ROS generation in MAC-T cells, hinting that HT has important effect against LPS-induced oxidative stress. Several lines of evidence have described that ROS overexpression LPS-induced in turn produces cytokines release [56]. Well in line with literature our study showed increased expression of TNF-α, IL-1β and IL-6 one and six hours after LPS-stimulation. The 10-μM and 25-μM HT treatment significantly prevent the increase of TNF-α, IL-1β and IL-6 LPS-induced. Several evidence suggest that HT is significantly able to improve endogenous antioxidant defense mechanisms by modulating transcription factors such as the Nrf2 [56,57]. Nrf2 is an important regulator of antioxidant signaling. It binds the epoxy chloropropane Kelch sample related protein-1 (Keap1) under homeostatic conditions. When the oxidative stress occurs, in cytoplasm the complex of Nrf2 and Keap1 is dissociated, and Nrf2 enters the nucleus [58]. Once translocated in the nucleus, Nrf2 binds to antioxidant response elements (ARE), promoting the transcriptional activation of metabolizing enzymes/antioxidant proteins enzymes (superoxide dismutase (SOD), Catalase (CAT), glutathione peroxidase (GSH-Px)) in phase II [59,60]. ROS produced by redox reactions can activate Nrf2 by promoting disulfide formation with Keap1 [61]. Therefore, the Nrf2–ARE signaling pathway is the main mechanism for defense against oxidative stress in cells by activating phase II metabolic enzymes/antioxidant protein enzymes [62].Our results showed upregulated the Nrf2 expression after one six hours from LPS-stimulation and HT treatment at bot concentrations (10 μM and 25 μM) increased its expression, confirming that HT antioxidant mechanism is mediated by the Nrf2 pathway. To further confirm the mechanism of the protective effect of HT we evaluated the mRNA expression of several antioxidant enzymes. In particular, our study displayed reduced heme oxygenase-1 (HO-1), NAD(P)H: quinone oxidoreductase (NQO-1), thioredoxin reductase 1 (Txnrd1) mRNA expression after LPS-stimulation. These antioxidant enzymes are phase II detoxifying proteins that provide intracellular defensive mechanism [14]. Our data displayed that the mRNA expressions of these antioxidant genes were upregulated in MAC-T cells treated with HT, suggesting that HO-1, NQO-1 and Txnrd1 are involved in the antioxidant mechanism of HT. These findings are well in line with precedent papers showing that HT reduces antioxidant enzymes [29].

One of the main functions of the bovine mammary epithelial cells is lactation. It involves prolactin, a hormone secreted by the anterior pituitary gland [63]. During lactogenesis and pregnancy prolactin has a crucial role in the mammary gland [64,65]. MAC-T cells stimulated by prolactin together with hydrocortisone, retinoic acid and insulin secret more β-casein [45,46,66]. Mammary epithelial cell differentiation is identified by the activation of genes coding for milk proteins [67]. Bovine mastitis causing cellular damage leads to disruption in lactation. We evaluated the protective effect of HT on this peculiar cell function evaluating the α-casein S1, α-casein S2 and β-casein mRNA levels [68,69]. LPS-stimulation induced the downregulation on the casein genes (α-casein S1, α-casein S2 and β-casein). HT increased the expression levels of three casein isoforms, indicating that protective effect of HT also preserves an important cell functionality.

## 5. Conclusions

Our results demonstrate that LPS challenge in MAC-T cell leads to an important increase in oxidative stress and inflammatory response, process that in differentiated MAC-T cells cause a significative impairment in cell functionality such as lactation. Our results showed also that the treatment with the well-known natural molecule HT, has a significantly protective effects. Together with the decrease of oxidative stress the inhibition of proinflammatory cytokines increase is a fundamental step in antagonizing the pathologic process and therefore in preventing or blocking the progression of bovine mastitis. Furthermore, HT treatment was able to significantly improve the cell antioxidant defense. Finally, this protective action of HT is traduced in an important cell functionality such as milk production. These results open a natural good prospective for the use of HT in treatment and prevention of bovine mastitis.

## Supplementary Materials

The following are available online at https://www.mdpi.com/2076-3921/9/8/693/s1, Table S1: Effects of HT on cell viability MTT assay, Table S2: Effects of HT on inflammation and oxidative stress in MAC-T cells stimulated with LPS for 1 h, Table S3: Effects of HT on inflammation and oxidative stress in MAC-T cells stimulated with LPS for 6 h, Table S4: Effects of HT on casein isoforms in differentiated MAC-T cells.
