# Supplementary material for: Effects of Hydroxytyrosol against Lipopolysaccharide-Induced Inflammation and Oxidative Stress in Bovine Mammary Epithelial Cells: A Natural Therapeutic Tool for Bovine Mastitis
Source: Antioxidants (Basel). 2020 Aug 3;9(8):693. doi: 10.3390/antiox9080693 (PMC7464001; doi:10.3390/antiox9080693)
Supplement: Supplementary file 1 [file antioxidants-09-00693-s001.pdf]

Table 1. Effects of HT on cell viability MTT assay.

	**LPS - / HT -**	**LPS +/HT -**	**LPS +/ HT 10μM**	**LPS +/ HT 25μM**
Cell viability	100 ± 0	99.2 ± 0.37	99 ± 0.44	96.8 ± 1.77

Table 2. Effects of HT on inflammation and oxidative stress in MAC-T cells stimulated with LPS for 1 hour

	**LPS - / HT -**	**LPS +/ HT-**	**LPS +/ HT 10μM**	**LPS +/ HT 25μM**
ROS	100 ± 0	282 ± 8.60	227.4 ± 19.02	211.4 ± 8.87
GSH	0.95 ± 0.02	0.5 ± 0.05	0.7 ± 0.04	0.78 ± 0.05
HO-1	1 ± 0	0.82 ± 0.05	1.18 ± 0.08	1.22 ± 0.07
NQO-1	1 ± 0	0.78 ± 0.06	1.17 ± 0.07	1.38 ± 0.13
Txnrd1	1 ± 0	0.61 ± 0.04	0.99 ± 0.09	1.06 ± 0.14
NOX-1	1 ± 0	2.76 ± 0.11	2.28 ± 0.08	1.98 ± 0.12
TNF-α	450 ± 97.47	3060 ± 163.1	2160 ± 271.3	1880 ± 276.4
IL1-β	118 ± 34.26	2214 ± 154.9	1650 ± 120.4	1400 ± 138.7
IL-6	170 ± 30	3840 ± 120.8	2760 ± 317.2	2720 ± 171.5

ROS, reactive oxigen species; GSH, glutathione peroxidase; HO-1, heme oxygenase-1; NQO-1, NAD(P)H quinone oxidoreductase-1;NOX-1, NADPH oxidase 1; Txnrd1,thioredoxin reductase 1;TNF-α,tumor necrosis factor α; IL1-β,interleukin 1-β; IL-6, interleukin 6.

Table 3. Effects of HT on inflammation and oxidative stress in MAC-T cells stimulated with LPS for 6 hours.

	**LPS - / HT -**	**LPS +/ HT-**	**LPS +/ HT 10μM**	**LPS +/ HT 25μM**
ROS	100 ± 0	321 ± 14.18	245 ± 14.49	200.2 ± 6
GSH	0.96 ± 0.02	0.4 ± 0.05	0.74 ± 0.06	0.84 ± 0.06
HO-1	1 ± 0	0.7 ± 0.08	1.2 ± 0.04	1.56 ± 0.07
NQO-1	1 ± 0	0.59 ± 0.08	1.19 ± 0.13	1.48 ± 0.08
Txnrd1	1 ± 0	0.53 ± 0.06	1.09 ± 0.09	1.2 ± 0.13
NOX-1	1 ± 0	2.74 ± 0.10	2.18 ± 0.08	1.78 ± 0.10
TNF-α	270 ± 86.02	3876 ± 85.88	2092 ± 92.27	1608 ± 174.3
IL1-β	118 ± 34.26	2988 ± 63.98	1842 ± 105.4	1288 ± 151
IL-6	170 ± 30	3074 ± 119.6	2179 ± 104.9	1572 ± 192.8

ROS, reactive oxigen species; GSH, glutathione peroxidase; HO-1, heme oxigenase 1; NQO-1,NAD(P)H quinone oxidoreductase-1 ;NOX-1, NADPH oxidase 1; Txnrd1,thioredoxin reductase 1;TNF-α,tumor necrosis factor α; IL1-β,interleukin 1-β; IL-6, interleukin 6.

Table 4. Effects of HT on casein isoforms in differentiated MAC-T cells

	No differentiation	Differentiation **LPS - / HT-**	Differentiation **LPS** +/ **HT** -	Differentiation **LPS** +/ **HT 10**μM	Differentiation LPS +/ HT 25μM
**α casein S1**	0.26 ± 0.05	1 ± 0	0.37 ± 0.07	0.75 ± 0.08	1.01 ± 0.06
**α casein S2**	0.26 ± 0.05	1 ± 0	0.34 ± 0.06	0.71 ± 0.12	1.07 ± 0.08
**β casein**	0.39 ± 0.05	1 ± 0	0.40 ± 0.08	0.90 ± 0.09	1.18 ± 0.09
